# Management of chemotherapy-associated febrile neutropenia

**DOI:** 10.1038/sj.bjc.6605272

**Published:** 2009-09-15

**Authors:** D Cameron

**Affiliations:** 1 NCRN Coordinating Centre, University of Leeds, MacMillan Wing, Fairbairn House, 71–75 Clarendon Road, Leeds LS2 9PH, UK

**Keywords:** febrile neutropenia, chemotherapy, local protocols, rapid referral, management

## Abstract

The development of febrile neutropenia during a course of chemotherapy is not only a life-threatening complication, it can also lead to a decision to reduce chemotherapy intensity in subsequent treatment cycles, thus putting patient outcomes at risk. Although there are strategies available for the primary prevention of febrile neutropenia, these are not widely used in the UK management of breast cancer. It is, therefore, paramount to have a well thought out and rigorously implemented care protocol for febrile neutropenia, involving patients, family/carers and health-care professionals in both primary and secondary care, to ensure early detection and effective management.

Febrile neutropenia is a serious side effect of many forms of chemotherapy (Aapro *et al*, 2006). It is associated with significant morbidity and mortality, and can lead to a decision to reduce or delay subsequent chemotherapy doses, which can have implications for treatment efficacy (Aapro *et al*, 2006). The risk of developing febrile neutropenia depends on the degree and duration of chemotherapy-induced neutropenia and on a number of patient factors, including age, comorbidity and serum albumin levels (Bodey *et al*, 1966; Meza *et al*, 2002; Lyman *et al*, 2005; Aapro *et al*, 2006).

The definition of febrile neutropenia, together with its possible consequences, is discussed elsewhere in this supplement (Krell and Jones, 2009). Similarly, elsewhere in this supplement, Kelly and Wheatley address the important question of how, and when, primary prevention is appropriate for patients with breast cancer (Kelly and Wheatley, 2009). However, primary prevention through the use of granulocyte colony-stimulating factors (G-CSF) is not commonly recommended in protocols for the management of breast cancer in the United Kingdom, and a significant proportion of patients undergoing chemotherapy for breast cancer go on to develop febrile neutropenia (Leonard *et al*, 2003), making its management an important consideration. This is particularly so in some of the taxane-based adjuvant chemotherapy regimens (Martin *et al*, 2005; Smith *et al*, 2006).

## Importance of local protocols

Effective local strategies for diagnosing and managing febrile neutropenia are essential components of breast cancer services (NCEPOD, 2008). Unfortunately, such strategies are prey to several potential stumbling blocks, as summarised in Table 1.

A survey reported in 2008 by the National Confidential Enquiry into Patient Outcome and Death (NCEPOD), looking at the care delivered to patients who died within 30 days of chemotherapy, identified several common failings in the management of febrile neutropenia (NCEPOD, 2008). Serious shortcomings were evident in patient education/awareness, health-care professional education/awareness and the availability and implementation of clear protocols for patient assessment, treatment and hospital admission (NCEPOD, 2008).

The NCEPOD report emphasises the need for rapid referral and assessment structures for potential febrile neutropenia to be in place, and for accessibility to all patients at risk. The models used may vary from centre to centre. However, the important feature is that all concerned, from the patient and their relatives, to primary and secondary care staff, are aware of the agreed procedure and management guidelines.

## What should patients look out for?

The first challenge in the diagnosis of febrile neutropenia is to make sure that patients will recognise signs suggesting that they are seriously ill – and take the necessary action.

It is essential to inform all patients receiving chemotherapy for breast cancer about the risk of febrile neutropenia, and to explain what to look out for, before they start their treatment. Typical signs include a temperature higher than 37.5°C, flu-like symptoms, mouth ulcers or a sore mouth that prevents eating (Malik *et al*, 2001). Less commonly with many of the regimens used in the treatment of breast cancer, patients may also have uncontrolled diarrhoea/vomiting, and uncontrolled nose bleeds or bleeding from the gums. Patients should also be made aware that they might not feel hot – indeed, those most at risk sometimes feel cold, or may merely describe systemic malaise/pain (Malik *et al*, 2001).

It is important to indicate to patients and carers, depending on the specific chemotherapy regimen, the days in the treatment cycle when neutrophil counts are likely to be at their lowest and the risk of febrile neutropenia is considered to be greatest. For example, many regimens have nadirs between days 10 and 14 (Hall *et al*, 2005), but docetaxel monotherapy tends to result in severe neutropenia several days earlier, on days 5–9 (Chan *et al*, 1999). However, timing should not be overemphasised, as the side effect can present outside of the typical days. This is particularly important when a patient is switching from anthracycline to docetaxel (as would be the case on the FEC-T (5-fluorouracil+epirubicin+cyclophosphamide followed by docetaxel) regimen often used in clinical practice), when it may be helpful to draw attention to the comparatively early neutrophil nadir associated with the latter drug and the potential, therefore, for cumulative risk (Head *et al*, 2008).

## Who should patients call, and when?

**Figure 1:**
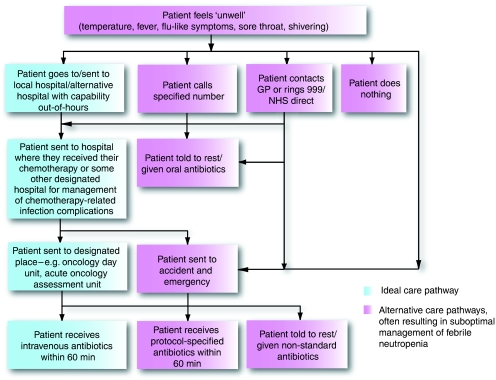
Gold standard care pathway for patient with febrile neutropenia.

The next challenge in the diagnosis and management of febrile neutropenia is to make sure the patient's symptoms are communicated appropriately and effectively, so that they enter the necessary care pathway (Figure 1).

One major pitfall is the reticence that many patients have towards ‘troubling the busy doctor’, particularly outside of surgery hours, and it is important to stress the need to seek help as soon as signs of possible febrile neutropenia develop.

Some chemotherapy teams, particularly those managing leukaemia, which carries an especially high risk of treatment-induced infection (Gillis *et al*, 1996), instruct patients to make direct contact with their chemotherapy service in the event of symptoms suggesting febrile neutropenia. However, equivalent advice for patients with solid tumours could swamp oncology staff in some services, and the approach taken varies across the country. For example, some teams ask patients to make direct contact with the chemotherapy service within clinic hours, and/or an on-call senior nurse outside of the normal working weekday; some instruct patients to contact emergency primary care services, whatever the day or time. The latter strategy carries the risk that the busy on-call GP will not get the full picture from the patient and will fail to recognise the severity of the risk, which also applies to patients visiting A&E with symptoms of febrile neutropenia.

The potential time lag from presentation to treatment bears a serious risk – as antibiotics should be administered within 60 min of any signs and symptoms of febrile neutropenia developing. Lack of communication between the treating physician at A&E and the patient's oncology department can lead to insufficient knowledge of the patient's case and history; this issue could be resolved by implementing communication protocols that ensure that the patient's oncology team is fully notified of any treatment that their patient has received. In light of these concerns, several chemotherapy services offer patients a back-up contact point if they feel unhappy with the input from community services.

## What should be assessed initially?

The initial assessment is clinical, beginning with the patient's vital signs, including the consciousness level, respiration, circulation and temperature.

Any chemotherapy patient who is clinically septic should be regarded as a medical emergency. If the assessment is taking place in primary care, it is essential for the GP to be aware that the patient is undergoing chemotherapy, and to have a low threshold for referral to hospital. To optimise communication at this stage, many chemotherapy services give patients an information sheet, outlining details of their chemotherapy regimen, advice on the signs of febrile neutropenia and guidance on when and where patients who may need treatment should be referred.

## Where should the patient be referred?

A patient on conventional chemotherapy who is well and afebrile does not need to be referred to hospital. However, if a patient on chemotherapy is febrile (e.g., over 38°C, or over 37.5°C for at least 1 h), or subthermic and at risk of being neutropenic, it is important to follow locally agreed management guidelines.

It is essential that all parties likely to be involved in the care of patients receiving chemotherapy – including health-care professionals in the community – are aware of the local guidelines and follow them. For example, many chemotherapy services clearly stipulate that patients should not be referred to A&E, but directly to the haematology or oncology inpatient ward/team, depending on the underlying cancer. However, even when such a policy is in place, it remains paramount to keep the local A&E staff informed and educated about management policies for patients who may have chemotherapy-induced febrile neutropenia. A&E departments may well receive these patients, despite local policy. For example, some patients will self-refer, and an individual who is very septic and/or unconscious may be brought to the department directly after a 999 call-out.

Referral to A&E presents a particular challenge to the optimum management of such patients. Compared with other people arriving in the department, a profoundly neutropenic and septic patient may look ‘well’, and there is a risk that their condition may deteriorate when they are left to await their turn in triage.

In light of this risk, and in line with the NCEPOD report on deaths after chemotherapy, all A&E departments should be aware of the local policy on the management of neutropenic sepsis, and copies of the policy should be easily available to all health professionals on duty (NCEPOD, 2008). Moreover, there should be ready access to staff who are familiar with the neutropenic sepsis policy, and antibiotics specified by the policy should be kept in the A&E department.

Importantly, the oncology team must be made aware of any patient on chemotherapy reporting and receiving treatment for neutropenic sepsis at A&E, as future cycles of chemotherapy may need to be delayed, dose adjusted or supported with prophylactic G-CSF or antibiotics in the interests of patient safety.

## How should the patient be managed?

On referral to hospital, rapid treatment of all patients with febrile neutropenia is paramount to prevent life-threatening deterioration of the minority who have a serious infection.

It is essential to have an agreed local policy on the standard investigations and interventions. These are not decisions that should be left in the hands of the junior doctor on duty (NCEPOD, 2008).

Full examination should include the mouth and perineum, and the basic blood tests required include full blood count, C-reactive protein, urea and electrolytes, creatinine, liver function, glucose, calcium and albumin (Malik *et al*, 2001; Hughes *et al*, 2002). Peripheral and, where appropriate, central venous blood cultures should be taken and examined for fungi and bacteria. Additional tests can be ordered as clinically appropriate, with a low threshold for stool culture (Malik *et al*, 2001; Hughes *et al*, 2002).

Most departments will have a policy for swabs, clotted serum for antibody titres, urinalysis and chest X-ray. Note, however, that the time required to organise and obtain an X-ray should not be allowed to delay urgent blood tests and the commencement of treatment. Indeed, any high-risk patient who needs to be taken off the ward should be escorted to allow continual clinical monitoring.

It is advisable to establish intravenous access at an early stage in case the patient subsequently develops shock (Malik *et al*, 2001). It is also sensible to commence intravenous fluids (unless contraindicated) when the full clinical picture is being assessed.

One key question is whether to commence antibiotics before the full blood count and biochemistry are known. This will depend on local practice and the severity of the clinical picture. For example, although local guidelines recommend the use of aminoglycosides, it may be prudent to wait until the patient's renal function is known. However, the NCEPOD report clearly emphasises the importance of rapid intervention if patients satisfy the criteria for febrile neutropenia, and recommends the empirical use of intravenous antibiotic therapy while the bacteriology results are awaited (NCEPOD, 2008).

## Admit or not?

It is a widespread practice to admit all patients with a diagnosis of febrile neutropenia, usually to a ward area where there is expertise in the management of the condition. Some district general hospitals admit such patients under the general medical team, but even some small hospitals have a designated area for the management of febrile neutropenia.

However, there is at least one randomised study that has shown no significant difference in mortality or morbidity between patients managed in hospital and those managed as outpatients, as long as a number of key criteria are met (Malik *et al*, 1995). These criteria include the lack of any significant systemic upset or comorbidity, and the use of daily monitoring (Moores, 2007). Few hospitals in the United Kingdom have implemented an outpatient approach to the management of febrile neutropenia (Ziglam *et al*, 2007), as such a policy would require changes in service delivery, including the provision of a demarcated day bed area for rapid assessment of new patients, a robust 24-h on-call system, a clear mechanism for admitting patients who deteriorate and dedicated staff for the daily review of current neutropenic patients and suspected cases. Clearly, the health-care professional who makes the initial assessment would need to be competent to decide who does or does not need to be admitted.

## Which type(s) of antibiotic?

The choice of which antibiotic(s) to use needs to be established as a local policy, in consultation with the clinical teams managing patients on chemotherapy, and the microbiologists who monitor local patterns of infection and resistance.

### First line

A combination of an aminoglycoside and a broad-spectrum antibiotic was established as the standard first-line therapy of febrile neutropenia in a series of randomised clinical trials from the European Organization for Research and Treatment of Cancer (Cometta and Glauser, 1996). However, there are data suggesting that broad-spectrum antibiotic monotherapy can be effective, particularly in low-risk patients (usually defined as those without comorbidity, organ dysfunction and localised or deep-seated sites of infection, who are normotensive and whose neutropenia is expected to be brief) (Karthaus *et al*, 1998; Chamorey *et al*, 2004; NWCN, 2007). Similarly, there are at least two randomised trials that have shown that combination oral antibiotics are at least as effective as standard intravenous combinations (Freifeld *et al*, 1999; Kern *et al*, 1999).

In some protocols, the presence of an indwelling device, such as a Hickman line, influences the choice of first-line antibiotic therapy, as such a device can change the distribution of likely causative organisms (Boland *et al*, 2003).

### Second line

Most policies suggest changing to a second-line antibiotic if the patient remains febrile for 48 h, or earlier if the patient deteriorates or positive blood cultures are obtained (Hoy, 2009). As with first-line antibiotics, it is important that agreed local policies are followed, along with expert clinical judgement.

## Is colony-stimulating therapy needed?

Again, local guidelines should set out the indications for using G-CSF during treatment of neutropenia.

Uncomplicated, short-duration neutropenia should not need routine G-CSF support, even if the patient is febrile. However, many local policies advocate the treatment if the patient is clinically septic and/or hypotensive, expected to have neutropenia of long duration (as with certain chemotherapeutic regimens for haematological diseases), or has organ dysfunction (NWCN, 2007). It is not good practice to wait and see if the patient struggles to recover from the neutropenic event; it is far better to identify those patients at greater risk and intervene early, even given the current absence of randomised data showing that this approach improves survival.

## Should patients be transferred to intensive care?

If a patient with a neutropenic event is considered sufficiently ill to warrant intensive care, then the transfer is almost always appropriate. One exception might be the patient who is being treated in the palliative setting who has clear evidence of refractory disease with little possibility of effective alternative systemic therapy. However, just because a patient does not have a curable disease, intensive care should not be ruled out. Ideally, such difficult decisions will be rendered unnecessary through previous discussions with the patient about their end-of-life care and by swift, appropriate management of febrile neutropenia.

## What are the implications for subsequent chemotherapy?

The implications of a neutropenic event for further chemotherapy depend very much on the treatment intent. Where the treatment is palliative, there is an important balance to be struck between, on the one hand, toxicity and loss of quality of life, and on the other, tumour shrinkage, life prolongation and improved quality of life. In most cases, this will mean that it is in the patient's best interest to have a delay in the next cycle of chemotherapy to allow time for a full recovery, and then a modification of the dose or regimen to reduce the risk of further neutropenic fever/sepsis. The use of secondary prophylactic G-CSF is an alternative, but one that is not widely used in palliative care in the United Kingdom.

In contrast, where the treatment is curative – adjuvant or neo-adjuvant – many clinicians would argue that it is important to maintain the chemotherapy dose and schedule. In this situation, although a delay may be needed to allow for reasonable recovery of the neutrophil levels, every effort should be made to get the subsequent cycles of chemotherapy completed on time and at the full dose, which may require secondary prophylaxis with G-CSF.

The decision whether or not to reduce or delay the next adjuvant chemotherapy dose is rendered more complex if the patient has suffered a genuinely life-threatening episode of neutropenia. A neutropenic death during adjuvant chemotherapy is a tragedy that should be avoided wherever possible – hence the potential argument for dose delay or reduction. However, it is also important to ensure effective treatment of the cancer, or the patient may survive febrile neutropenia only to die later from metastatic disease. The risk of neutropenia in subsequent cycles is reduced by the use of primary prophylaxis of febrile neutropenia, and this should be considered where indicated by the risk associated with the regimen and individual patient factors (Gafter-Gvili *et al*, 2005; Aapro *et al*, 2006; Smith *et al*, 2006; Kelly and Wheatley, 2009).

## What can we learn from practice?

Audit of the circumstances surrounding cases of febrile neutropenia, particularly those resulting in death or severe morbidity, highlights good and poor practice, and provides pointers for improving care for patients undergoing chemotherapy.

The NCEPOD survey found that only about half of the hospitals conducted audits of neutropenic sepsis, and that only 16% of the deaths within 30 days of systemic anti-cancer therapy were discussed at morbidity and mortality meetings (NCEPOD, 2008). The report recommends a formal discussion of all such deaths, a regular audit of all cases of neutropenic sepsis following chemotherapy, and the inclusion of protected time for audit in consultants’ job plans (NCEPOD, 2008).

## Conclusion

Febrile neutropenia is a common and serious complication of chemotherapy, and hence it is important to have locally agreed protocols that are well publicised across primary and secondary care, and subject to regular review. Regular audit, in-depth discussion of deaths and serious morbidity associated with febrile neutropenia, and active liaison with health-care professionals in the primary and secondary care services are important to ensure that all patients with possible febrile neutropenia are assessed and treated appropriately, consistently, safely and rapidly.

## Figures and Tables

**Table 1:** Potential pitfalls (and solutions) in the diagnosis and management of febrile neutropenia in women undergoing chemotherapy for breast cancer (NCEPOD, 2008; NCAG, 2009)

**Potential pitfall**	**Solution/s**
Patient unaware of the significance of early symptoms of febrile neutropenia	Patient education Clear take-home information
Patient reluctant to seek help out of hours	Patient education Clear take-home information
Patient contacts GP, who fails to recognise the magnitude of the risk	Information and liaison across primary and secondary care Patient-held information card to guide GP Back-up contact point for patients who are not sure their GP has responded appropriately
Patient referred (or self-referred) to A&E, and not prioritised in triage	Provision of education and information for A&E staff Copy of local protocol available in every A&E department Access to staff who are familiar with the policy Appropriate antibiotics available in A&E
Delays in symptom assessment and treatment during transfer to radiology	Patient accompanied and monitored when taken off the ward
Delayed delivery of antibiotic therapy when awaiting investigation results	Empirical use of antibiotic therapy
